# Acute dengue virus 2 infection in Gabonese patients is associated with an early innate immune response, including strong interferon alpha production

**DOI:** 10.1186/1471-2334-10-356

**Published:** 2010-12-17

**Authors:** Pierre Becquart, Nadia Wauquier, Dieudonné Nkoghe, Angélique Ndjoyi-Mbiguino, Cindy Padilla, Marc Souris, Eric M Leroy

**Affiliations:** 1 Centre International de Recherches Médicales de Franceville, BP 769 Franceville, Gabon; 2 UMR 264 MIVEGEC, Institut de Recherche pour le Développement (IRD)/Centre National pour la Recherche Scientifique/Université Montpellier I, Montpellier, France; 3 Institut National de la Santé et de la Recherche Médicale UMR-S 945, Laboratoire d'Immunité et Infection, Université Pierre et Marie Curie Paris 6, Paris, France; 4 Université des Sciences de la Santé, Libreville, Gabon; 5 UMR 190 Emergence des pathologies virales/IRD, Marseille, France; 6 CVVD, Faculty of Science, Mahidol University, Salaya, Thailand

## Abstract

**Background:**

Dengue is now a leading cause of morbidity and mortality throughout the tropics. We conducted the first *ex vivo *study of dengue fever (DF) in African patients infected during the first Gabonese dengue virus 2 (DENV-2) outbreak in 2007, in order to investigate cytokine production, including the antiviral cytokine IFN-α, reported to be a potent inhibitor of DENV replication *in vitro*.

**Methods:**

Levels of 50 cytokines, chemokines and growth factors were measured in plasma from 36 patients with DENV-2 infection, and in uninfected controls, using Luminex multiplex technology. The results were interpreted according to the day of sampling after symptom onset. PBMC from six patients were also studied for T lymphocyte cell surface marker expression by flow cytometry.

**Results:**

Acute DENV-2 infection elicited high levels of several pro-inflammatory cytokines (IL-6 and IL-17), chemokines (MIF, RANTES, IP-10 and MCP-1) and growth factors (G-CSF, GM-CSF and VEGF-A). We also observed high levels of IFN-α for the first time in adult DF patients, and CD4+ and CD8+ T cell activation at symptom onset.

**Conclusion:**

Acute DENV-2 infection in African patients elicits a strong innate response involving IFN-α production, as well as an adaptive immune response.

## Background

Dengue virus (DENV), of which four serotypes have been identified (DENV-1 through DENV-4), is a single-stranded positive-sense RNA virus belonging to the genus *Flavivirus *of the *Flaviviridae *family. DENV generally causes an acute self-limited illness known as classic dengue fever (DF), lasting 5-7 days [1]. Symptoms include high fever, headache, retro-orbital headache, myalgia, arthralgia, abdominal pain, nausea and vomiting. A minority of patients (less than 3%) develop dengue hemorrhagic fever (DHF) or dengue shock syndrome (DSS), the most severe form. Dengue is the most frequent human vector-borne viral disease. Until the 1960s, DENV was mainly restricted to tropical and subtropical regions, especially south-east Asia, but it has now spread to South Asia, South and Central America, the Caribbean, and Africa. DENV is transmitted by peridomestic female mosquitoes of the genus *Aedes *[2]. *Ae. aegyptii*, present in most endemic regions [3], was considered to be the main vector of DENV. Nevertheless, *Ae. albopictus *was suspected of being the main vector of DENV during major epidemics [4] and was shown to be the only vector during simultaneous outbreaks of chikungunya and DENV-2 infection occurring in Gabon in 2007 [5]. Two-fifths of the world population are at risk, and an estimated 50 to 100 million cases of DF occur each year worldwide. About 500 000 people develop DHF and about 20 000 deaths occur, mainly among children under 15 years of age. The incidence of DENV infection has risen more than 30-fold in the past 50 years [6,7]. Despite the major health and economic impact of this disease, there is currently no vaccine and no specific treatment.

Numerous studies have explored the cytokine response to DENV. The cellular IFN system is the mainstay of host defenses during the first days of infection, a phase during which the intensity of viral replication determines clinical outcome [8]. *In vitro*, DENV infection of human cells can be inhibited by pretreatment with IFN-α/β and IFN-γ [9], which inhibit translation of the viral RNA input strand [10]. Interestingly, DENV-2-infected monocyte-derived dendritic cells *in vitro *fail to prime T cells, due to the lack of IFN-α/β produced in those cells after infection [11]. The importance of the IFN-α response *in vivo *is illustrated by the increased lethality of mouse-adapted DENV-2 virus when administered by intraperitoneal injection to IFN-α/β and γ receptor knockout mice [12]. Little is known of human plasma IFN-α concentrations during the acute phase of the illness. One study showed elevated IFN-α plasma levels shortly after symptom onset in DF children [13]. Proinflammatory cytokines such as TNF-α, IFN-γ, Il-6, Il-18 and MIF are also known to be involved during the acute phase of the illness [14-18], and many chemokines involved in leukocyte recruitment to sites of infection, such as IL-8, IP-10 and MCP-1, are produced during inflammation [19-23]. Primary cultured human monocytes infected by DENV-2 produce proinflammatory cytokines such as IFN-α, TNF-α and IL-6 [24,25], and PBMC infected by DENV-2 *in vitro *produce pro-inflammatory cytokines and other soluble mediators, including TNF-α, IFN-γ, IL-2, Il-4, IL-5, Il-6 and Il-10 [26]. Evidence of T cell activation has also been observed, with a rise in the percentage of CD4+ and CD8+ T lymphocytes expressing CD69, an early activation marker, reported in children with acute DF [27].

In severe forms (DHF/DSS), after a normal acute phase, capillary permeability increases abruptly and the resulting plasma leakage can lead to circulatory shock and death. The severity of DENV infection seems to be due more to disproportionate inflammatory cytokine production than to direct viral effects [28-30]. Antibody-dependent enhancement (ADE) has also been implicated, based on observations that re-infection by a different viral serotype increases the risk of severe dengue [31]. In this case, rapid activation of cross-reactive DENV-specific memory T cells generated during primary DENV infection appears to trigger strong production of proinflammatory cytokines such as IFN-γ, TNF-α and Il-6, that can directly damage vascular endothelial cells, further resulting in plasma leakage [29,32]. DENV-specific T cells may have a dual role, both helping to clear the virus and causing bystander tissue damage. Finally, DENV, like many other viruses, has evolved molecular mechanisms to circumvent IFN-mediated responses [33] and expression of DENV non-structural proteins (NS5B, NS4B, NS4A, NS2A) by infected cells has been shown to disrupt the IFN-α/β signalling pathway [34-38].

Most *ex vivo *studies have involved patients in endemic regions of Asia and America, and the serotype was often not clearly identified. The incidence of DENV has increased in Africa since 2000, especially in central Africa, indicating a change in DENV epidemiology in this region [5,39,40]. We conducted the first *ex vivo *study of African DF patients infected during the first Gabonese DENV outbreak of 2007. The patients had probably never encountered DENV previously, and were therefore unlikely to have developed specific immunity. In order to investigate cytokine expression, and especially the role of IFN-α, during the acute phase of DENV infection in adults, we used Luminex multiplex technology to measure the levels of 50 soluble factors, including cytokines never or rarely studied in this setting. We also explored T lymphocyte responses during the first days after symptom onset.

## Methods

### Patients and samples

Between March and August 2007, simultaneous chikungunya and dengue outbreaks occurred in Gabon, initially in the capital city Libreville [5]. Outbreaks subsequently occurred in several small towns along the route to northern Gabon and Cameroon, causing about 20,000 cases. During the course of the outbreak, 773 blood samples were collected during the first week after symptom onset from febrile patients who visited healthcare centers in Libreville and other Gabonese towns. The patients' demographic characteristics, clinical features and symptom duration were recorded. Blood was drawn into EDTA tubes (Becton Dickinson) and transported on ice daily to the virology laboratory of Libreville faculty of medicine. The tubes were centrifuged for 10 min at 2000 *g *at room temperature, and plasma was recovered, aliquoted and stored at -80°C. Peripheral blood mononuclear cells (PBMC) were isolated by standard Histopaque density centrifugation if sufficient blood was available. PBMC were resuspended in fetal calf serum (FCS) (Invitrogen) with 10% dimethyl sulfoxide (Merck) in Cryovials (Nunc), kept at -80°C overnight, then transferred to liquid nitrogen and transported weekly to the Franceville International Medical Research Center (CIRMF).

Samples were tested at CIRMF for DENV and chikungunya virus RNA by using TaqMan quantitative reverse transcription-PCR (qRT-PCR) technology. Among the 773 patients, 275 (35.6%) and 54 (7.0%) were positive for chikungunya virus and DENV, respectively. Using a dengue serotype-specific qRT-PCR assay, we showed that all the patients were infected by serotype 2 strains (DENV-2) [5]. A quantified synthetic DENV RNA transcript kindly provided by the University of the Mediterranean (Prof. X. De Lamballerie) was used as standard for the quantification of DENV-2 RNA in positive samples. All amplifications were performed in duplicate.

No severe cases of dengue infection (DHF, DSS) were observed during this epidemic. The DENV-2-positive samples were negative for various arbovirus RNA genomes (Chikungunya, West Nile, yellow fever and Rift Valley fever viruses), as tested by Taqman qRT-PCR, and also for West-Nile virus antibodies, as tested with an ELISA method.

Thirty control plasma samples were randomly selected among 4349 samples collected from healthy individuals throughout Gabon during a previous study [41]. These individuals were themselves randomly selected among the Gabonese rural population, excluding children and elderly persons (more than 65 years).

PBMC from five randomly selected healthy volunteers (40% male; mean age 29 years, range 25-32 years) were also used as controls.

### Ethical considerations

The study was approved by the Gabonese Ministry of Health (authorization N°000006 MSP/SG), and written informed consent was granted by all the patients and healthy controls.

### Multiplex analysis

Concentrations of 50 cytokines, chemokines and growth factors were measured in plasma by means of Luminex technology (Bio-Rad). Two kits -- the Bio-plex human cytokine 27-plex assay and the Bio-plex human cytokine 23-plex assay (Bio-Rad) -- were used, as recommended by the manufacturer. The following cytokines were measured: interleukin-1β (IL-1β), IL-1 receptor antagonist (IL-1Ra), IL-2, IL-4, IL-5, IL-6, IL-7, IL-8, IL-9, IL-10, IL-12p70, IL-13, IL-15, IL-17, eotaxin, basic fibroblast growth factor (FGF-basic), granulocyte colony-stimulating factor (G-CSF), granulocyte macrophage colony-stimulating factor (GM-CSF), IFN-γ, IP-10, monocyte chemoattractant protein 1 (MCP-1), macrophage inflammatory protein-1α (MIP-1α), MIP-1β, platelet-derived growth factor-ββ (PDGF-ββ), regulated-on-activation normal T-cell expressed and secreted (RANTES), tumor necrosis factor-α (TNF-α), and vascular endothelial growth factor (VEGF) in the 27-plex assay; and Il-1α, IL-2Rα, Il-3, Il-12p40, IL-16, IL-18, CTACK (or CCL27), GRO-alpha (or CXCL1), hepatocyte growth factor (HGF), intracellular adhesion molecule 1 (ICAM-1), IFN-α2, leukemia inhibitory factor (LIF), MCP-3 (or CCL7), macrophage colony-stimulating factor (M-CSF), monokine induced by interferon-gamma (MIG or CXCL9), nerve growth factor-β (NGF-β), stem cell factor (SCF), stem cell growth factor β (SCGF-β), SDF-1α (or CXCL12), tumor necrosis factor beta (TNF-β), tumor necrosis factor beta (TRAIL) and VCAM-1 in the 23-plex assay. Briefly, 25 μL of plasma was diluted 1:4 in Bioplex human sample diluent and incubated with anti-cytokine antibody-coupled beads for one hour at room temperature. Between each step the complexes were washed three times in wash buffer (Bio-Rad), using a vacuum manifold. The beads were then incubated with a biotinylated detector antibody for one hour before incubation with streptavidin-phycoerythrin for 30 minutes. Finally, the complexes were resuspended in 125 μL of detection buffer and 200 beads were counted with a Luminex 200™ device (Bio-Rad). Final concentrations were calculated from the mean fluorescence intensity and expressed in pg/mL.

### Flow cytometry

Patient and control PBMC were thawed, washed thrice in RPMI 1640 with 1% penicillin/streptomycin, and resuspended at a final density of 2 × 10^6 ^cells/mL in RPMI with 10% FCS and 1% penicillin/streptomycin for 18 hours at 37°C. Prior to staining, cells were washed in RPMI then distributed at a final density of 1 × 10^6 ^cells/mL in 0.1 mL of Isoflow (Beckman Coulter) in three tubes containing monoclonal antibodies, as follows: tube 1: CD3-FITC, CD4-PE, CD69-PC5 and CD8-PC7; tube 2: CD3-FITC, CD4-PE, HLADR-PC5 and CD8-PC7; tube 3 CD3-FITC, CD95-PE, CD4-PC5 and CD8-PC7 (Beckman Coulter). After staining for 20 minutes at room temperature and the addition of 200 μL of Isoflow, 100 000 events were captured on a FC500 cytometer (Beckman Coulter) and analyzed with CXP software (Beckman Coulter).

### Statistical analysis

Student's *t *test was used to compare normally distributed cytokine concentrations between the patients and controls, while the Mann Whitney Wilcoxon test was used for non normally distributed data. Normality was tested with the Shapiro-Wilk test. P values below 0.01 were considered to indicate significant differences (to take account of multitesting for the groups of cytokines, the type I risk was reduced from 0.05 to 0.01 using Bonferroni correction). STATA 10.0 (College Station, Texas USA) and SavGIS 9.05 software (IRD, France) was used for all analyses.

## Results

### DENV-2 viral load

Viral load (VL) was measured in plasma by real-time quantitative PCR in DENV-2-infected patients. Mean VL was 1.2 × 10^6 ^± 4.4 × 10^1 ^cDNA copies/mL (2.7 × 10^4 ^to 5.1 × 10^7 ^cDNA copies/mL). VL did not vary significantly with age, sex or the day of sampling after symptom onset (data not shown).

### Plasma cytokine, chemokine and growth factor levels

Soluble factor concentrations were measured in 36 DENV-2-infected patients (40% male; mean age 31 years, range 18-66 years) from whom single blood samples were collected between D0 and D11 after symptom onset, and 30 randomly selected uninfected and healthy individuals (60% male; mean age 47 years, range 30-65 years). Levels in the patients' samples were further interpreted according to the time (in days) after symptom onset (D0). One patient was sampled on day 0, seven on D1, ten on D2, nine on D3, two on D4, three on day 5, two on D7 and two on D11. The 23-plex assay could not be applied to samples obtained from four patients on D7 and D8, owing to insufficient sample volume.

**Figure 1:**
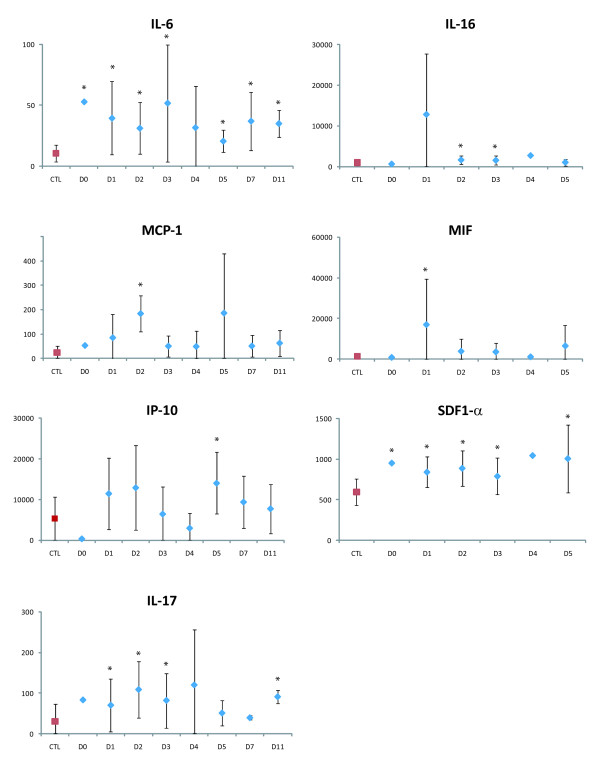
**Pro-inflammatory cytokines and chemokines upregulated in plasma of DF patients **. Levels are expressed in pg/mL, according to the day of sampling after symptom onset (*p < 0.01). Mean control (CTL) levels are shown in red. The vertical bars represent the standard deviation. One patient was sampled on day 0, seven on D1, ten on D2, nine on D3, two on D4, three on day 5, two on D7 and two on D11.

**Figure 2:**
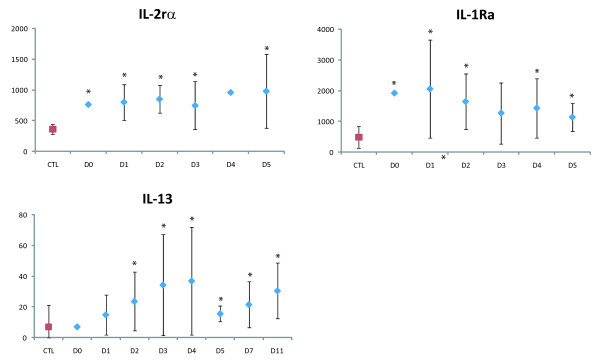
**Anti-inflammatory cytokines upregulated in plasma of DF patients **. Levels are expressed in pg/mL, according to the day of sampling after symptom onset (*p < 0.01). Mean control (CTL) levels are shown in red. The vertical bars represent the standard deviation. One patient was sampled on day 0, seven on D1, ten on D2, nine on D3, two on D4, three on day 5, two on D7 and two on D11.

**Figure 3:**
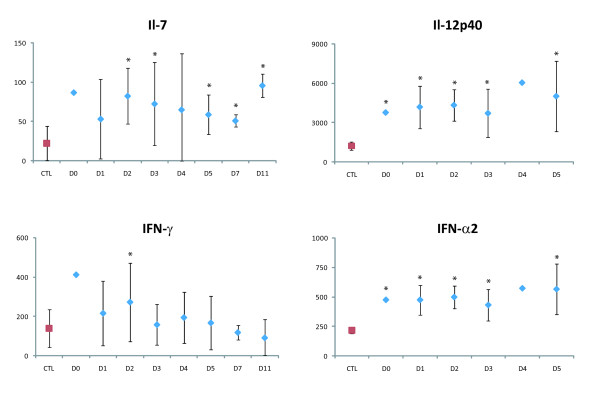
**Cytokines upregulated in plasma of DF patients**. Levels are expressed in pg/mL, according to the day of sampling after symptom onset (*p < 0.01). Mean control (CTL) levels are shown in red. The vertical bars represent the standard deviation. One patient was sampled on day 0, seven on D1, ten on D2, nine on D3, two on D4, three on day 5, two on D7 and two on D11.

**Figure 4:**
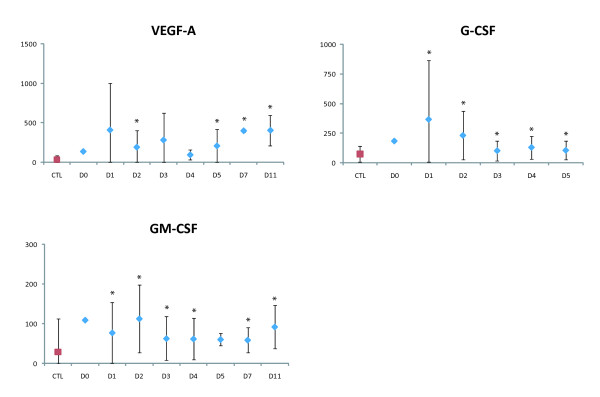
**Plasma growth factors upregulated in plasma of DF patients **. Levels are expressed in pg/mL, according to the day of sampling after symptom onset (*p < 0.01). Mean control (CTL) levels are shown in red. The vertical bars represent the standard deviation. One patient was sampled on day 0, seven on D1, ten on D2, nine on D3, two on D4, three on day 5, two on D7 and two on D11.

Relative to the controls, the patients had significantly elevated levels (p < 0.01) of growth factors (G-CSF, GM-CSF and VEGF-A), pro-inflammatory and antiviral cytokines (IL-6, Il-17 and IFN-α2), anti-inflammatory cytokines (IL-1Ra, IL-2rα and Il-13), chemokines (Il-16, MCP-1, IP-10, SDF-1α, MIF and RANTES), and cytokines associated with adaptive responses (Il-7, Il-12p40 and IFN-γ) (Figure 1, Figure 2, Figure 3 and Figure 4).

During the first days of symptoms, levels of IL-1Ra, IL-2rα, Il-6, IL-7, IL-12p40, IL-13, IFN-α2, VEGF-A and SDF-1α were significantly higher in the patients than in the controls (Figure 1, Figure 2, Figure 3 and Figure 4). IFN-α2 levels exceeded 400 pg/mL throughout the acute phase in 20/26 patients (controls: 217 ± 28 pg/mL). Il-16 and Il-17 levels were significantly elevated from D2 to D3 (Figure 1), and G-CSF and GM-CSF levels from D1 to D5 and D1 to D11, respectively (Figure 4). IFN-γ was elevated (>200 pg/mL) in the only patient sampled on D0 (p < 0.01), and in most of the patients sampled on D1 (5/8) and D2 (6/10; p < 0.01) (Figure 3). MIF was elevated on D1, MCP-1 on D2, and IP-10 on D5 (p < 0.01) (Figure 1).

Levels of ICAM-1, VCAM-1 and RANTES were above the working range of the assay in the patients but not in the controls (data not shown).

**Figure 5:**
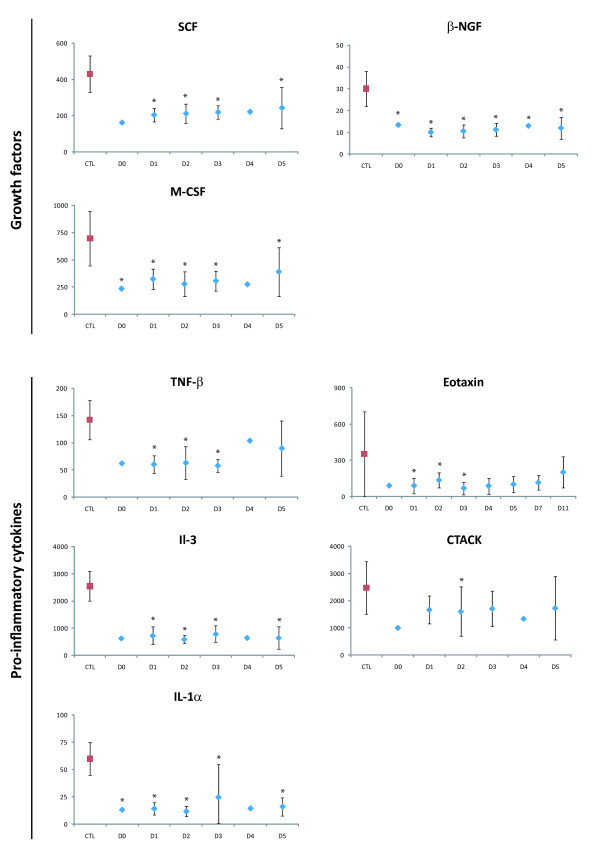
**Pro-inflammatory cytokines and growth factors downregulated in plasma of DF patients **. Levels are expressed in pg/mL, according to the day of sampling after symptom onset (*p < 0.01). Mean control (CTL) levels are shown in red. The vertical bars represent the standard deviation. One patient was sampled on day 0, seven on D1, ten on D2, nine on D3, two on D4, three on day 5, two on D7 and two on D11.

Levels of IL-3, TNF-β, Il-1α, eotaxin, CTACK, M-CSF, SCF and β-NGF were lower in the patients than in the controls (Figure 5).

There were no significant differences between the patients and controls regarding the concentrations of IL-1β, Il-4, IL-5, IL-8, IL-9, IL-10, IL-15, IL-18, TNF-α, MIP-1α, MIP-1β, MCP-3, Il-12p70, FGF-basic, HGF, PDGF-ββ, LIF, MIG, SCGF-β or TRAIL (see Additional file 1).

### Flow cytometry

PBMC from 6 patients (34% male; mean age 34 years, range 20-47 years) and 5 controls were studied for T cell surface markers (Table 1). The mean age and the male/female gender ratio were not different between the two groups (p > 0.7; data not shown). Positive gating for lymphocytes based on forward and side scatter was followed by CD3+ CD4+ and CD3+ CD8+ gating. Statistical tests were not used, owing to the limited number of patients (one patient on D0, one patient on D1, two patients on D2, two patients on D3). The percentage of CD3+ CD8+ T cells was higher on D0 in the patients than in the controls (41% *versus *16%), while the percentage of CD3+ CD4+ lymphocytes was stable during the acute phase.

**Table 1:** T lymphocyte phenotypes in DF patients during the first days after symptom onset.

	CD3+ CD4+	CD3+ CD8+	CD3+ CD4+			CD3+ CD8+		
			
			CD69+	HLA-DR+	CD95+	CD69+	HLA-DR+	CD95+
Controls (N = 5)	40	16	9	4	3	23	8	11
D0 (N = 1)	32	41	6	14	31	7	38	3
D1 (N = 1)	41	17	29	12	25	51	6	2
D2 (N = 2)	44	17	8	4	25	14	16	13
D3 (N = 2)	48	11	14	3	40	18	14	10

Results are expressed as percentages.

Expression of the activation markers CD69 and HLA-DR was further analyzed. CD3+ CD4+ T cells reached a maximum activation on D0 and D1, as shown by a peak in CD69 expression on D1 (29% *versus *9% in controls) and in HLA-DR expression on D0 (13% *versus *4% in controls) and D1 (12% *versus *4% in controls). The percentage of CD3+ CD8+ CD69+ lymphocytes was highest on D1 (51% *versus *23% in controls), while the percentage of CD3+ CD8+ HLA-DR+ lymphocytes was highest on D0 (38% *versus *8% in controls).

CD95 expression on CD3+ CD4+ T lymphocytes was elevated in all the patients on D0 to D3 (31% on D0, 25% on D1 and D2, and 39% on D3, *versus *2% in the controls). In contrast, percentage of CD3+ CD8+ CD95+ T lymphocytes was lower on D0 and D1 (3% on D0 and 2% on D1) than in the controls (11%), while no difference was observed on D2 or D3.

## Discussion

This is the first study of the innate immune response to DENV in an African population. One of the most significant results of this study is the sustained elevated levels of IFN-α2 throughout the acute phase of the illness in all DENV-2-infected patients. IFN-α, mainly produced by dendritic cells and macrophages [42], is a first line of host defense, limiting viral replication. In addition to its antiviral affects, IFN-α induces the expression of major histocompatibility complex class I molecules, enhancing antigen presentation and thereby triggering the acquired immune response. IFN also induces antigen-specific CD8+ cell responses and chemokine production, leading to activation of lymphocytes and monocytes and their recruitment to sites of inflammation [43]. A previous study of Thai children aged 5-14 years with DF showed elevated IFN-α levels only on D1 and D3 after fever onset [13]. This slight discrepancy with our findings might be due to immunological immaturity, detection of different IFN-α subtypes [44], infection by different DENV serotypes, assay sensitivity and specificity, or differences in the choice of standards. Our results confirm, in DENV-2-infected adults, the potential role of IFN-α in DENV infection, as shown *in vitro *and in animal studies [9,10,12]. Studies exploring the relationship between viral load and the clinical severity of dengue infection have given conflicting results [8,45-47]. We found no correlation between virus load (measured by qRT-PCR) and IFN-α levels (r^2 ^< 0.4; data not shown). This could be explained by the absence of severe cases of DENV infection, the consistently high IFN-α levels in all DENV-2 infected patients, the small size of the study population, and infection by a different DENV serotype. A more recent study in Taiwan suggested that patients with secondary DEN-2 infections mount an altered immune response with lower IFN-α levels, 3-7 days after symptom onset, associated with the onset of DHF [48]. This suggests that the earlier proinflammatory reaction associated with IFN-α may be critical for the pathogenesis of secondary DENV-2 infections. Interestingly, in another study, IFN-α levels fell after the peak in viremia, one day before defervescence in Thai children aged 6 months to 14 years with secondary DENV-3 infection [46]. In our study, more than 85% of DENV-2 infected patients (data not shown) still had fever 5 days after symptom onset, and high IFN-α levels persisted throughout the acute phase. IFN-α levels could not be measured on the day of defervescence.

The innate response to DENV in these patients was also characterized by the production of proinflammatory chemokines and growth factors (Il-6, RANTES, IP-10, MCP-1, G-CSF, GM-CSF and VEGF-A). One originality of this study is that, contrary to previous reports, soluble factors level were measured throughout the acute phase [14,18,21-23,49,50]. Chemokines are produced by activated monocytes and macrophages, which are known DENV target cells [51]. Chemokines orchestrate leukocyte recruitment to sites of viral infection and play a key role in inflammatory responses [52]. Growth factors such as G-CSF and GM-CSF may contribute to the differentiation of myeloid progenitors and resting monocytes (GM-CSF) [53] or granulocyte progenitors (G-CSF) [54]. VEGF, a factor produced by many cells, including macrophages [55] and endothelial cells [56], is the most potent permeability-enhancing cytokine and seems to play an important role in the plasma leakage observed in DHF [57]. Interestingly, we observed elevated IL-17 levels during the first days after symptom onset. Il-17 is the signature cytokine of the newly described T helper 17 (Th-17) cell population [58] and has been proposed as the founding member of a new subclass of proinflammatory cytokines involved in dengue, eliciting cytokines such as Il-6, Il-8, MCP-1, GRO-α and TNF-α [59]. Levels of the proinflammatory cytokine TNF-α were not altered, in keeping with previous studies [15,18,60]. In contrast, two studies have shown TNF-α elevation in patients in Venezuela [14] and India [61]. Differences in population characteristics or prior immune status may explain this discrepancy with our results.

We also observed elevated levels of the anti-inflammatory cytokines IL-2Rα, IL-1Ra and IL-13 in most of our patients, throughout the acute phase. It is generally assumed that circulating IL-1Ra diffuses into tissues and influences the local Il-1Ra:Il-1 ratio, modulating a variety of Il-1-related immune and inflammatory responses [62]. The elevated IL-13 levels observed here are in keeping with previous findings [17]. Il-13 is produced by T lymphocytes and inhibits the production of pro-inflammatory cytokines by activated monocytes [63]. High levels of these anti-inflammatory cytokines may contribute to limiting bystander tissue damage that could otherwise occur during prolonged inflammation.

IFN-γ and Il-7 promote adaptive immunity [64-66], and the elevated levels observed here therefore suggest the involvement of cellular responses during DENV infection. Levels of IFN-γ, which is mainly produced by activated professional antigen-presenting cells, T lymphocytes and NK cells [64], also point to T cell activation. High levels of IFN-γ were also observed in previous studies [15,18,61,67,68]. These results are supported by the increased expression of activation markers (CD69 and HLA-DR) on CD4+ and CD8+ T cells at symptom onset. A previous study showed an increase in the percentage of CD69-expressing CD4+ and CD8+ T cells in Thai children within 72 h after fever onset [27].

Surprisingly, we observed CD95 upregulation on CD3+ CD4+ T cells during the first four days after symptom onset (16-40% of cells, compared to 2% in controls). In contrast, no differences in CD95 expression on CD3+ CD8+ T lymphocytes were observed. To our knowledge, this is the first study of the percentage of CD3+ CD4+ and CD8+ CD95+ T lymphocytes during the acute phase of DF. In a previous study, TUNEL staining of fragmented DNA showed that large numbers of PBMC (mostly CD8+ T lymphocytes) were undergoing apoptosis around the time of defervescence in DENV-1- or DENV-3-infected Thai children [69], and the degree of apoptosis correlated with disease severity and with the risk of DHF. After the virus has been cleared (after 1 to 7 days) and defervescence occurs [8], CD95/CD95 ligand-mediated apoptosis may be one mechanism promoting the death of activated T cells. However, owing to the small size of our panel (6 patients), larger investigations are required to further explore apoptosis of T lymphocyte subpopulations during dengue infection.

## Conclusions

This is the first study of the immune response to dengue virus in African patients, who had probably never previously encountered this pathogen. We observed high IFN-α plasma levels during the acute phase, supporting the results of *in vitro *and animal studies. Furthermore, we confirm in African patients that DENV-2 infection induces a strong innate response involving numerous pro-inflammatory factors, as well as an adaptive immune response involving CD4 and CD8 T cell activation. These findings raise the possibility that IFN-α administration might prevent progression to severe forms of dengue fever.

## Competing interests

The authors declare that they have no competing interests.

## Authors' contributions

PB and NW contributed equally to the study. PB and NW conceived and designed the experiments. PB and NW performed the experiments. PB, NW, EML, CP and MS analyzed the data. DN, ANM contributed reagents, materials and analytical tools. MS performed the statistical analysis. PB wrote the paper. All the authors have read and approved the final manuscript.

## Pre-publication history

The pre-publication history for this paper can be accessed here:

http://www.biomedcentral.com/1471-2334/10/356/prepub

## Supplementary Material

Additional file 1 **Cytokines, chemokines and growth factors unchanged in DF patients compared to controls (p > 0.05) throughout the acute phase**. Levels are expressed in pg/mL, according to the day of sampling after symptom onset. Mean control (CTL) levels are shown in red. The vertical bars represent the standard deviation. One patient was sampled on day 0, seven on D1, ten on D2, nine on D3, two on D4, three on day 5, two on D7 and two on D11. Click here for file
