# Supplementary figures and images for: Acute dengue virus 2 infection in Gabonese patients is associated with an early innate immune response, including strong interferon alpha production
Source: BMC Infect Dis. 2010 Dec 17;10:356. doi: 10.1186/1471-2334-10-356 (PMC3013083; doi:10.1186/1471-2334-10-356)

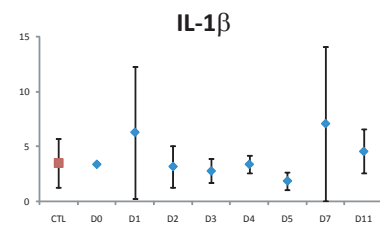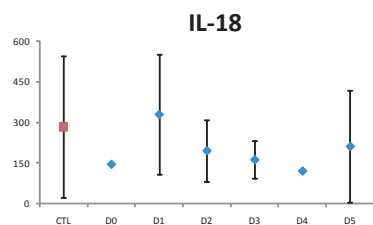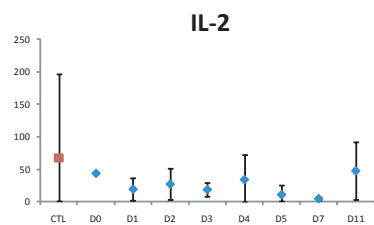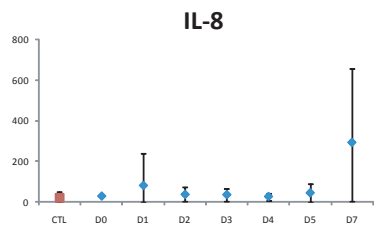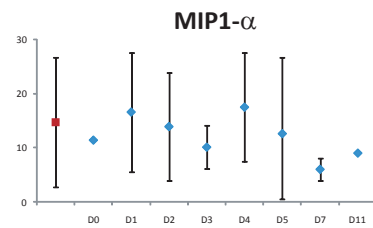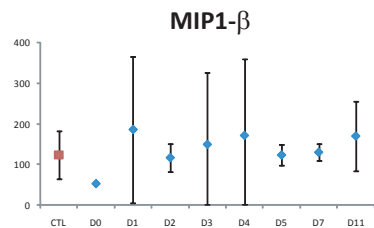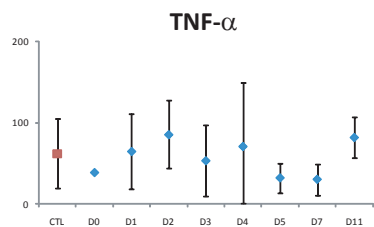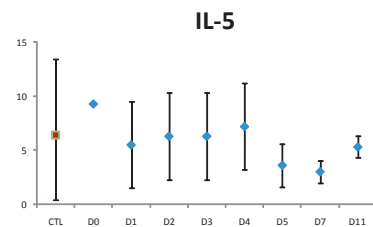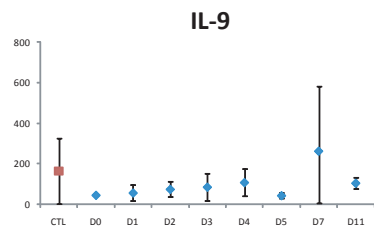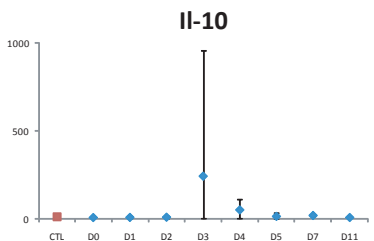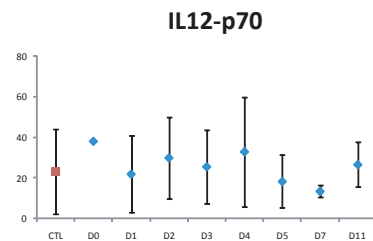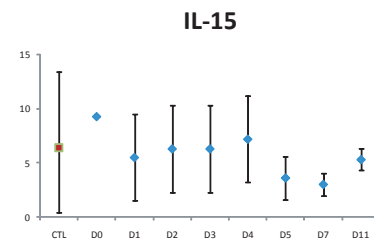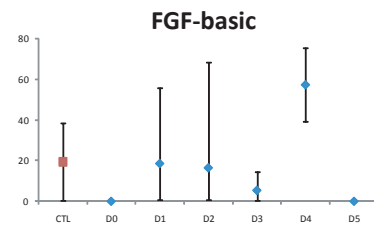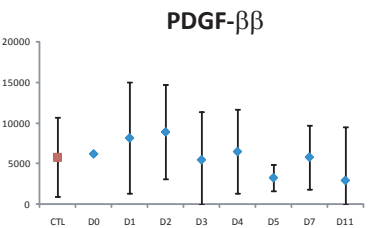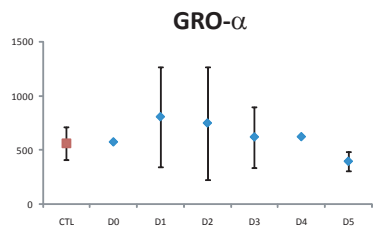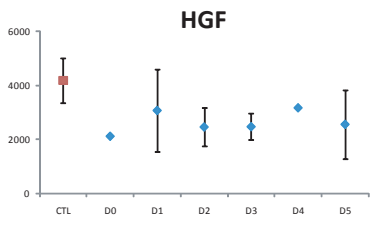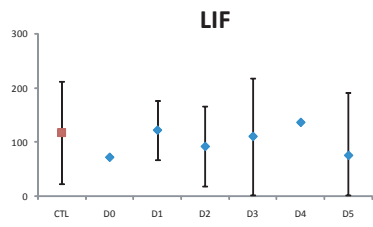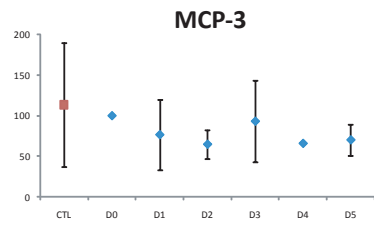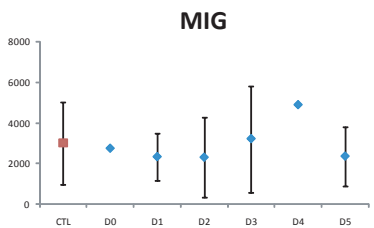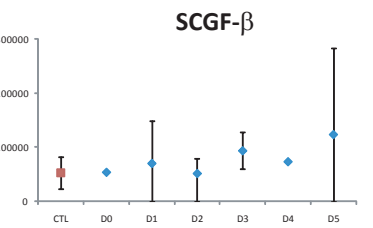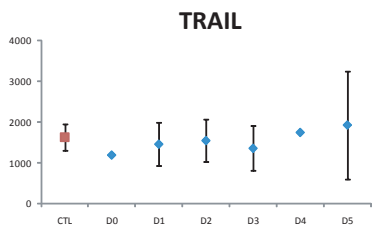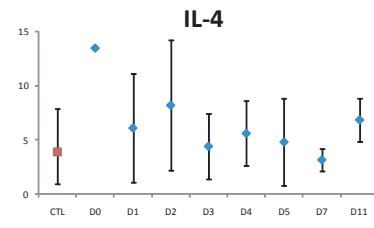

Supplement: Additional file 1 — Cytokines, chemokines and growth factors unchanged in DF patients compared to controls (p > 0.05) throughout the acute phase. Levels are expressed in pg/mL, according to the day of sampling after symptom onset. Mean control (CTL) levels are shown in red. The vertical bars represent the standard deviation. One patient was sampled on day 0, seven on D1, ten on D2, nine on D3, two on D4, three on day 5, two on D7 and two on D11. [file 1471-2334-10-356-S1.PDF]
